# The Efficiency Study of Graphene Synthesis on Copper Substrate via Chemical Vapor Deposition Method with Methanol Precursor

**DOI:** 10.3390/nano13061136

**Published:** 2023-03-22

**Authors:** Bohr-Ran Huang, Shang-Chao Hung, Yung-Shou Ho, Yi-Siou Chen, Wein-Duo Yang

**Affiliations:** 1 Graduate Institute of Electro-Optical Engineering, Department of Electronic Engineering, National Taiwan University of Science and Technology, Taipei 106, Taiwan; 2 Fuzhou Polytechnic, Fuzhou University City, Fuzhou 350108, China; 3 Intelligent Technology Research Centre, Fuzhou 350108, China; 4 Department of Applied Chemistry and Materials Science, Fooyin University, Kaohsiung 831, Taiwan; 5 Department of Chemical and Materials Engineering, National Kaohsiung University of Science and Technology, Kaohsiung 807, Taiwan

**Keywords:** graphene, methanol, copper substrate, chemical vapor deposition

## Abstract

Few-layer graphene was successfully synthesized on copper foil via chemical vapor deposition with methanol as a carbon source. This was confirmed by optical microscopy observation, Raman spectra measurement, I_2D_/I_G_ ratio calculation, and 2D-FWHM value comparisons. Monolayer graphene was also found in similar standard procedures, but it required higher growth temperature and longer time periods. The cost-efficient growth conditions for few-layer graphene are thoroughly discussed via TEM observation and AFM measurement. In addition, it has been confirmed that the growth period can be shortened by increasing growth temperature. With the H_2_ gas flow rate fixed at 15 sccm, few-layer graphene was synthesized at the lower growth temperature of 700 °C in 30 min, and at 900 °C growth temperature in only 5 min. Successful growth was also achieved without adding hydrogen gas flow; this is probably because H_2_ can be induced from the decomposition of methanol. Through further defects study of few-layer graphene via TEM observation and AFM measurement, we tried to find possible ways for efficiency and quality management in graphene synthesis in industrial applications. Lastly, we investigated graphene formation after pre-treatment with different gas compositions, and found that gas selection is a crucial factor for a successful synthesis.

## 1. Introduction

Graphene can be conducted as a kind of functional material for flexible and transparent devices due to its unique structures, excellent physical properties, and terrific mechanical and flexible characteristics, which result in lots of potential applications in the fields of biosensors [1,2], supercapacitors [3], liquid crystal devices [4], electronic devices [5], solar cell [6,7], catalysts [8], and even in energy storage and battery applications [9]. Owing to the sp^2^ bonds and π bond with carbon atoms tightly and regularly arranged in a single layer of hexagonal honeycomb-like, two-dimensional structures [10], the electrons in graphene can move freely which result in an excellent electrical conductivity and very high mobility at room temperature [11,12]. With this stable elastic lattice structure, the carbon atoms in monolayer graphene need not be rearranged to adapt to the external forces for elongation and bending [13], resulting in a superior thermal conductivity [14]. Transistors with graphene material can probably be stably operated at scales close to a single atom [15]. Monolayer graphene also exhibits excellent penetration in optical characteristics [16]. Therefore, graphene is an ideal transparent electrothermal conductor for making transparent electrode in organic photovoltaics [17] and heat dissipative coatings in light emitting diodes (LEDs) [18] for industrial applications instead of the indium tin oxide (ITO), fluoride-doped oxide (FTO), and aluminum oxide-doped (AZO) with advantages of being cheaper, thinner, and non-toxic.

The application of graphene composite and graphene-based inorganic nanocomposites currently has attracted more and more attention for enhancing the specific characteristic performance in various kinds of composite materials. For example, the cytotoxicity studies of biocompatibility with graphene/CNT hybrid silicone composites, exhibit high elasticity, good durability, low resistivity, and prominent electrical stability because of the few-layer graphene and CNTs ratio and hybrid filler loading [19]. Prior study of the supercapacitor with MnO_2_/graphene/Ni electrodes exhibits specific capacitance and mechanical stability improvement because of the buffer layer of graphene between MnO_2_ and Ni foams [20]. The field emission performance improvement study via the graphene aligned with the gradient on the substrate surface shows the field enhancement factor increasing and the turn-on field decreasing because of the graphene composition [21]. Another responsivity study of the heteroepitaxial interfaces with 2D graphene covered on 1D nanotubes as a hydrogen gas sensing layer shows the aggravation of composition effect with increasing graphene content because of the gas diffusion interactions between two-dimensional layered graphene structure and various geometries [22]. Moreover, the biosensors study for glucose detection shows the electrochemical interaction, whereas glucose oxidase (GOx) is adsorbing on laser-induced graphene electrodes because of its high chemical stability, excellent electrical conductivity, and high specific surface area [23]. These intensive studies found that the geometrical properties of graphene such as variations and fluctuations, could be the dominant factor of performance in not only biosensors and supercapacitors promotion, but also in field emission enhancement and gas sensitivity improvement. Very recently, twisted multilayer graphene has been synthesized and found its outstanding transport properties [24]. Complete demonstration of twisted phase, twist angles, and Moiré superlattices in band structures also has been realized [25]. Therefore, confirming the quality of graphene and the growth efficiency should be another focus of research because a number of monolayers (less than 10 layers) of graphene still exhibit 2D properties [26].

Graphene can be commonly prepared via solid phase methods such as mechanical exfoliation [27] and SiC epitaxial growth [28,29] or via liquid phase methods such as the chemical exfoliation method including chemical reducing graphene oxide [30,31] and intercalation stripping [32]. Graphene can be easily obtained by the mechanical exfoliation method but with time-consuming preparation and is hardly used in industry [33]. Excellent quality graphene can be obtained by the SiC epitaxial growth method with 4H-SiC or 6H-SiC used through a high-temperature and low-pressure process but with expensive materials and complex processes [34]. The chemical reduction method is relatively easy to prepare graphene, but defects exist caused by impurities in the process because the chemical functional groups stretch the graphene [35]. A large-area graphene synthesizing method currently used is chemical vapor deposition (CVD) which belongs to the gas phase method [36,37]. This method, which only requires a transition metal substrate and a carbon source, also can transfer to other substrates for industry use.

Graphene growth temperatures are dependent on two key factors of precursors and substrates. The layer number and quality of graphene are dependent on the catalytic activities and carbon solubilities of the catalysts used. Intensive works have reported graphene synthesis with polycrystalline nickel [38] and copper [39] foil/film as transition metal substrates instead of single crystal metals such as Pt [40], Ru [41], Mo [42], Ni, and Ir [43] because of the cost concern. Graphene can be synthesized with solid carbon sources instead of gaseous carbon sources such as methane, acetylene, and ethylene due to temperature and safety concerns, but the reaction process was more complicated because of the large molecular structures. Graphene synthesis via CVD with gaseous hydrocarbons as carbon sources is a state of the art mature technology, but the problems are fatalness, expense, and high-temperature considerations, which result in a demand to find alternative carbon sources that are low-risk, cheap, and easy-to-obtain. Consequently, graphene synthesized with liquid carbon sources such as alcohol or benzene rings have received attention recently based on the correlation of cost efficiency, safety, pressure, synthesis temperature, and environmental friendliness. However, fast carbon deposition with graphene synthesizing results in multilayer graphene generation mainly due to the benzene ring with six carbons. Therefore, methanol was selected as the carbon source instead of benzene rings in this study because the monolayer graphene synthesis did not need a large amount of carbon deposition. In addition, copper foil was used as the substrate in this study to possibly synthesize monolayer graphene in a low-pressure atmosphere due to the surface growth on copper with low solubility of carbon.

## 2. Experimental

The graphene synthesis equipment in this study is a self-assembled low-pressure chemical vapor deposition (CVD) system with a quartz tube (tube length: 100 cm, outer diameter: 5.5 cm, inner diameter: 4.5 cm), a tubular high-temperature furnace, a mechanical pumper, a dry pumper, and a steel cup. High-purity argon (Ar: 99.999%) was used as the carrier gas passing through the steel cup containing methanol, and then the methanol can be carried into the growth system as a carbon source. In the high-temperature experimental stage, the mechanical pump was used to pump the system pressure to 10^−2^ Torr, follow by a dry pump for graphene growth at the same fixed pressure to avoid the impact of the mechanical pump’s oil–gas backflushing to the system.

All the growth substrates were cut from copper foil (99.8%, Alfa Aesar, Karlsruhe, Germany),) with a thickness of 25 μm into many square pieces with a size of 0.5 × 0.5 cm^2^. At the beginning of the experiment, the copper foil in the crucible was placed into the quartz tube. Evacuating the system pressure to 10^−2^ Torr by using the mechanical pump followed by the dry pump with a flow rate of 200 sccm (20% H_2_/80% Ar) for 1 h to ensure the oxygen in the quartz tube was completely removed. For the growth temperature study, the tubular furnace with H_2_/Ar atmosphere (200 sccm) was heated to the desired growth temperature of 500 °C, 700 °C, 800 °C, 850 °C, 900 °C, and 950 °C, respectively, for 30 min. The copper oxide residue on the copper foil surface was then eliminated. After 30 min. of copper oxide residue elimination, the H_2_/Ar atmosphere flow rate was reduced from 200 sccm to 35 sccm with the composition of 15/20 sccm in the desired growth temperature. The methanol in the steel cup was carried into the system via the carrier gas of Ar for 30 min. of operation. For the gas composition study, the tubular furnace with H_2_/Ar atmosphere (200 sccm) was heated to the growth temperature of 900 °C for 30 min. After 30 min. of copper oxide residue elimination, the H_2_/Ar atmosphere flow rate was, respectively, reduced from 200 sccm to the desired composition with H_2_ of 0, 10, 15, 20 sccm, and Ar of 20 sccm as a carrier gas for methanol into the system for 30 min. of operation. For the growth period study, the tubular furnace with H_2_/Ar atmosphere (200 sccm) was heated to the growth temperature of 900 °C for 30 min. After 30 min. of copper oxide residue elimination, the H_2_/Ar atmosphere flow rate was reduced from 200 sccm to 35 sccm with a composition of 15/20 sccm. The methanol in the steel cup was carried into the system via the carrier gas of Ar for 30 min. of operation. At the end of the temperature, gas composition, and growth period experiment, the carbon source stopped supplying while accomplishing the graphene growth. The tubular furnace was cooled down to room temperature under the H_2_/Ar atmosphere flow rate of 200 sccm. The obtained samples were taken out of the tubular furnace after the system pressure was recovered to 1 pressure atmosphere and subjected to OM and Raman analysis.

Preliminary observations of all samples were made by using an upright optical microscope, also known as a metallographic microscope. The light source used is a mono-wavelength beam passed through a filter to reduce chromatic aberrations. The images of these microstructures are examined using an external computer and analysis software with an image processing function. The best identification rate of an optical microscope is 2000 Å, and the magnification is about 1000–1500 times. The quality of graphene and the number of layers were confirmed using Raman spectroscopy (Raman, Horiba HR550) with the excitation laser light source of 532 nm wavelength and laser spot width of ~2 μm, combined with an analysis device which is composed of an analytical light spectrometer and a low-temperature CCD. The Raman spectroscopic analysis can be performed with the test piece without special treatment. While performing, three different positions were measured in each sample. Every set of Raman data is the average value of two results. The number of layers and the quality of graphene can be analyzed from the peaks in the Raman spectrum. Transmission electron microscope, (TEM) (JEOL TEM-3010) and multi-functional scanning probe microscope, (Icon-AFM) (BRUKER Dimension Icon) were utilized for further observation. While the TEM samples and the AFM specimens were prepared, graphene was ultrasonically oscillated in anhydrous alcohol, and subjected to, respectively, a drop of evenly dispersed liquid on a carbon-coated copper grid, and on the Si substrate, followed by vacuum drying. AFM measurements were performed in the scan range of 5 μm × 5 μm × 5 nm with a resolution of X, Y < 1.0 nm, Z < 0.1 nm.

## 3. Results and Discussion

**Figure 1:**
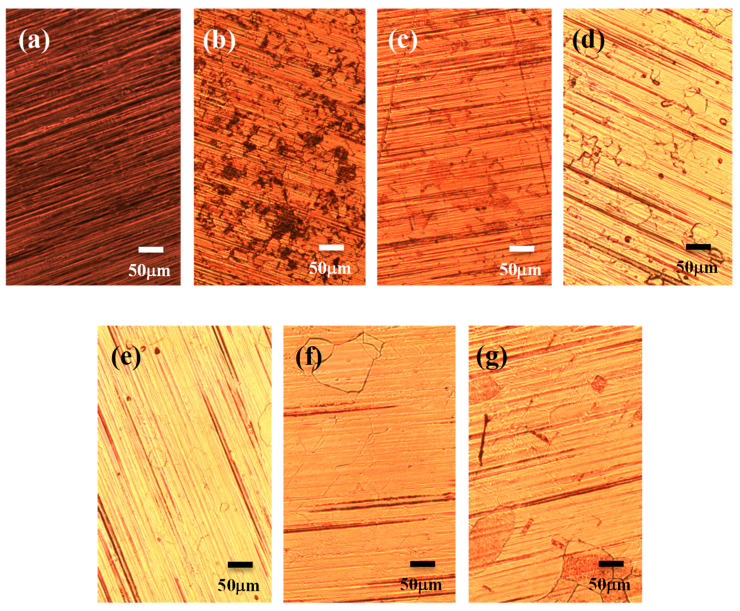
Optical micrographs of the (**a**) bare copper foil, and graphene grown in the atmosphere of 20 sccm Ar carrying methanol and 15 sccm H_2_, for 30 min. with temperatures of (**b**) 500 °C, (**c**) 700 °C, (**d**) 800 °C, (**e**) 850 °C, (**f**) 900 °C, (**g**) 950 °C, respectively.

Figure 1a shows the optical micrograph of the copper foil specimen. It is observed that the surface of the copper foil is covered with imprint marks produced in the preparation process, and the copper grains cannot be clearly seen. Figure 1b–g shows optical micrographs of the graphene grown on a copper foil substrate with 15 sccm hydrogen gas and 20 sccm Ar carrying methanol at growth temperatures of 500 °C, 700 °C, 800 °C, 850 °C, 900 °C, and 950 °C in the time period of 30 min., respectively. It can be found that the imprints on the surface of the copper foil were significantly reduced, and copper crystal grains were generated after the thermal process. The copper crystal grains increased as the growth temperature increased from 500 °C to 950 °C accordingly. To confirm the graphene quality corresponding to the growth temperatures, characterizations of the number of graphene layers by using Raman spectroscopy should be further investigated.

**Figure 2:**
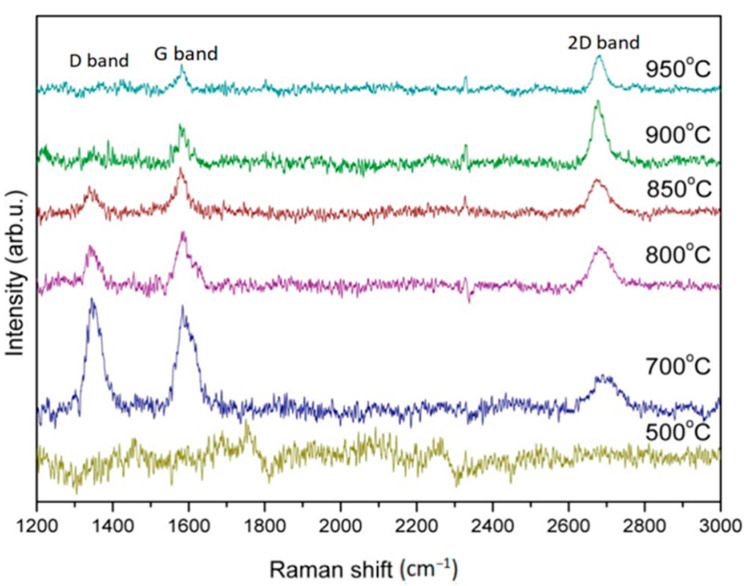
Raman patterns of graphene grown on copper foil substrate with 20 sccm Ar carrying methanol and 15 sccm H_2_ in the time period of 30 min at growth temperatures of 500 °C, 700 °C, 800 °C, 850 °C, 900 °C, 950 °C, respectively.

Figure 2 shows the Raman spectra patterns of graphene grown on a copper foil substrate with 20 sccm Ar carrying methanol and 15 sccm H_2_ at growth temperatures of 500 °C, 700 °C, 800 °C, 850 °C, 900 °C, and 950 °C in the time period of 30 min, respectively. Any graphene generated from the figure in the 500 °C growth temperature can not be observed. This result agrees with the report from Gadipelli et al. [44] because of the low growth temperature.

As is well known, the typical Raman spectra of graphene show the G band and 2D band at about 1500~1600 cm^−1^ and 2650~2700 cm^−1^, respectively. The number of graphene layers can be realized through the intensity ratio of these two peaks as I_2D_/I_G_. Moreover, the number of graphene layers can also be confirmed from the position of the 2D band. If the peak of 2D band showed a higher frequency of movement, a so-called blue-shift phenomenon, the graphene is a multi-layer graphene. Note that, if a D band is found at 1250~1350 cm^−1^ in the Raman spectrum, which is a defect peak, the graphene shows a poor quality because of the disordered graphene structures. In addition, monolayer graphene shows the magnitude of I_2D_/I_G_ ratio greater than 1.5. The number of graphene layers can also be determined by the 2D half width at half maximum (2D-FWHM) value. Monolayer graphene shows the magnitude of FWHM value around 30–36 cm^−1^. The magnitude of 2D-FWHM value greater than 36 cm^−1^ can be classified as double layers or few layers graphene.

The characteristic Raman spectra peaks of graphene in this step, respectively, show the D band, G band, and 2D band at ~1350, ~1580, and ~2700 cm^−1^, respectively, in the growth temperature of 700 °C. The I_2D_/I_G_ ratio and the 2D-FWHM value were calculated as about 0.33 and 66 cm^−1^, respectively, which proves that graphene can not only be synthesized at the temperature of 700 °C, but also a at few-layered properties. However, the defect peak of D band is obviously found with the I_D_/I_G_ ratio of about 1.07, indicating lots of defects exist. Gadipelli et al. reported that methanol can start decomposing at 700 °C to produce H_2_, CO, and CH_4_, and be completely decomposed at 800 °C resulting in the graphene synthesized with CH_4_ is usually at around 900~1000 °C. Therefore, the carbon source for the graphene synthesis in the temperature of 700 °C is hardly from the CH_4_. Both the oxygen atoms decomposed from CO, and H_2_ produced from methanol can be easily combined to form a little water and be taken out of the furnace tube by pumping. Therefore, the main source of carbon for synthesizing graphene in 700 °C could be probably from the CO. As the growth temperature increased in 800 °C, 850 °C, 900 °C, and 950 °C, the I_2D_/I_G_ were 0.80, 0.74, 1.60, 1.34, and the 2D-FWHMs were 60, 54, 30, and 36 cm^−1^, respectively. It is found that I_2D_/I_G_ gradually increased, but 2D-FWHMs gradually decreased, as increasing the growth temperature. But as the growth temperature increases above 900 °C, the I_2D_/I_G_ ratio decreases, and the 2D FWHMs increases slightly. The defect peak of D band is obviously found with the I_D_/I_G_ ratio of about 1.07, indicating lots of defects exist. Moreover, the D band decreased as the growth temperature increased, and the defect peak was hardly observed while the growth temperature was above 900 °C.

**Figure 3:**
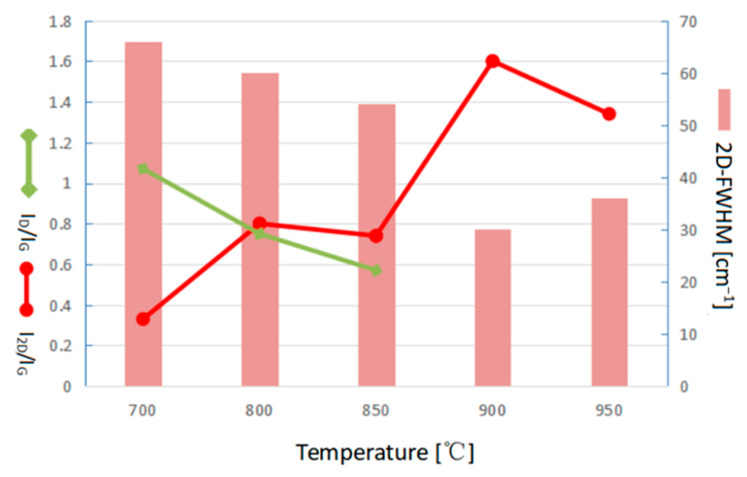
The comparation of I_2D_/I_G_ ratio, 2D-FWHM value, and I_D_/I_G_ ratio of Raman pattern corresponding to the growth temperature.

Figure 3 collectively plots the comparison of I_2D_/I_G_ ratio, 2D-FWHM, and I_D_/I_G_ ratio corresponding to the growth temperature. It can be clearly seen that the good quality of monolayer graphene was successfully synthesized at a temperature around 900 °C. In the graphene synthesis works using a gaseous carbon source, the growth temperature needs to be around 900–1000 °C [45,46,47,48,49]. In our experiment, graphene at a synthesizing temperature of 700 °C using Ar gas caring liquid methanol has been found in agrowth temperature lower than that of using a gaseous carbon source of about 100–200 °C. More works have also proved that graphene synthesized using a liquid carbon source can reduce the function temperature [50,51]. In addition, Guermoune et al. [52] also announced a graphene synthesis using methanol at 650 °C but with many defects, which agrees with our study.

It worth mentioning that Gadipelli et al. [44] reported a growth temperature of 900 °C for synthesizing graphene. The methanol was used as the carbon source without supporting hydrogen gas as the reducing gas but simply with the H_2_ decomposed from the thermal cracking of methanol as the reducing agent. Because methanol can play two roles in high-temperature decomposition, one is as the source of carbon, and the other is as the generation of OH radicals which can also inhibit the formation of amorphous carbon. Consequently, graphene can also be simply synthesized by using the H_2_ decomposed from the thermal pyrolysis of methanol as a reducing agent without directly adding hydrogen as a reducing gas. Hydrogen not only can reduce copper oxide and remove impurities on the surface of copper foil but also can restrain the formation of amorphous carbon [53]. Choubak et al. showed no evidence of graphene etching by purified ultrahigh purity (UHP)-grade hydrogen with the etching reaction of graphene films growing on copper foils at 825 °C and 500 m Torr [54]. Therefore, it is necessary to explore in detail the dependence between graphene growth and the flow rate of hydrogen gas.

**Figure 4:**
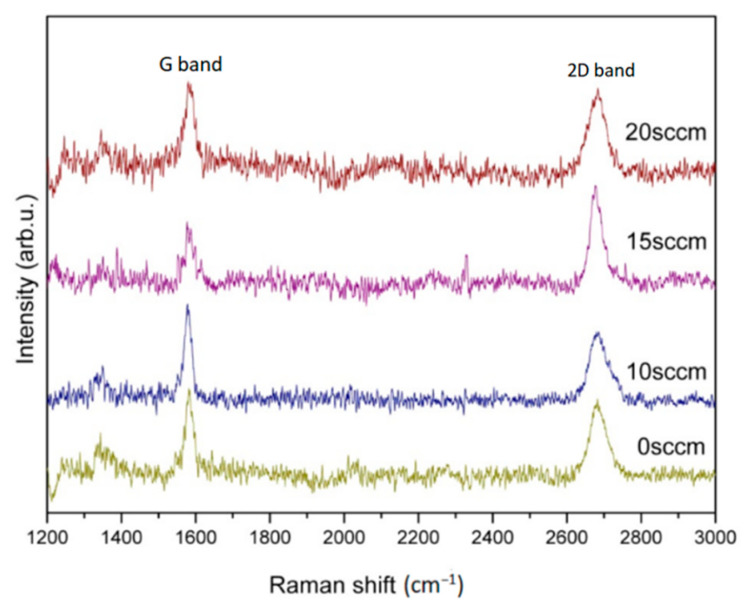
Raman spectra of graphene grown in Ar (20 sccm) with carbon source and various hydrogen flows rate (0, 10, 15, 20 sccm) at 900 °C for 30 min.

Figure 4 shows Raman spectra of graphene grown in Ar (20 sccm) with carbon source and various hydrogen flows rate (0, 10, 15, 20 sccm) at 900 °C for 30 min. Typical graphene peaks, G bands and 2D bands, can be, respectively, found at 1500~1600 cm^−1^ and 2650~2700 cm^−1^ on all specimens. Notably, the impurity peak of the D band cannot be found on all grown specimens indicating suitable parameters management on atmosphere compositions and temperatures. The I_2D_/I_G_ ratio and 2D-FWHM value of graphene grown in hydrogen flow rates of 0, 10, 15, and 20 sccm were calculated as 0.87, 0.71, 1.60, 0.9, and 48 cm^−1^, 54 cm^−1^, 30 cm^−1^, 54 cm^−1^, respectively. Both values were found to be returned after 15 sccm reached an extreme indicating a quality inversion.

**Figure 5:**
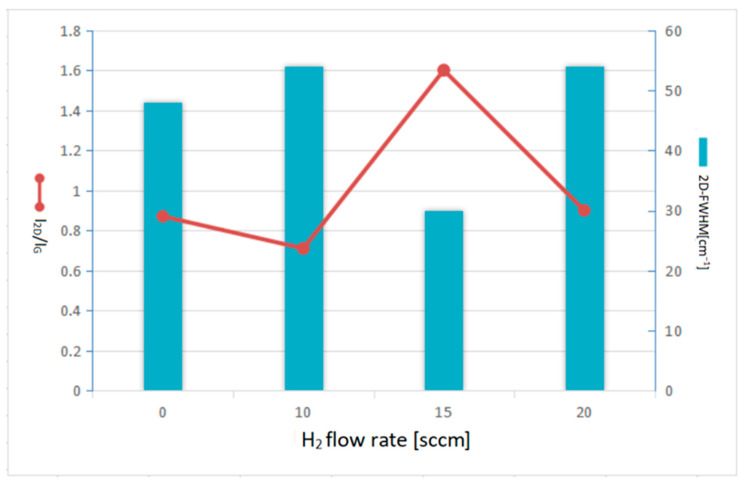
Relations of I_2D_/I_G_ ratio and 2D-FWHM value of peaks in Raman spectra corresponding to the hydrogen gas flows rate of 0, 10, 15, 20 sccm, respectively.

Figure 5 collectively plots the relation of I_2D_/I_G_ ratio, 2D-FWHM value, and I_D_/I_G_ ratio corresponding to the hydrogen gas flow rate (0, 10, 15, 20 sccm). It can be seen that a number of monolayer graphene explicated with 1.6 of I_2D_/I_G_ ratio and 30 of 2D-FWHM value has been successfully completed with a confirmation of hydrogen gas at 15 sccm flow rate in this stage of growth work. In addition, the number of graphene layers tended to be increased as the hydrogen gas flow gradually deviates from 15 sccm. This result proved that proper hydrogen flow rate is conducive to the few-layer graphene growth. Si et al. have reported that the influence of the hydrogen gas flow rate on carbon source in the graphene synthesis is that the number of graphene layers increased from monolayer to double layers as the hydrogen flow rate increased from 0 to 3 sccm [55]. Wang et al. have announced that larger CH_4_/H_2_ ratios cause larger sizes of graphene crystals [56]. In addition, based on the energy analysis, Li et al. calculated the dissociation of CH_4_ on graphene by using density functional theory (DFT) and found that the dissociation of CH_4_ into CH_3_ and H is a rate-determining step [57].

Since the time course of sample shipment is affected by the processing time. The cost of growth is also dependent on the uninterrupted supply of gas and thermal energy while synthesizing. Therefore, the growth time period is also one of the key factors of the production cost for the finished product. The next step should be to study the efficiency times of few-layer graphene synthesis.

**Figure 6:**
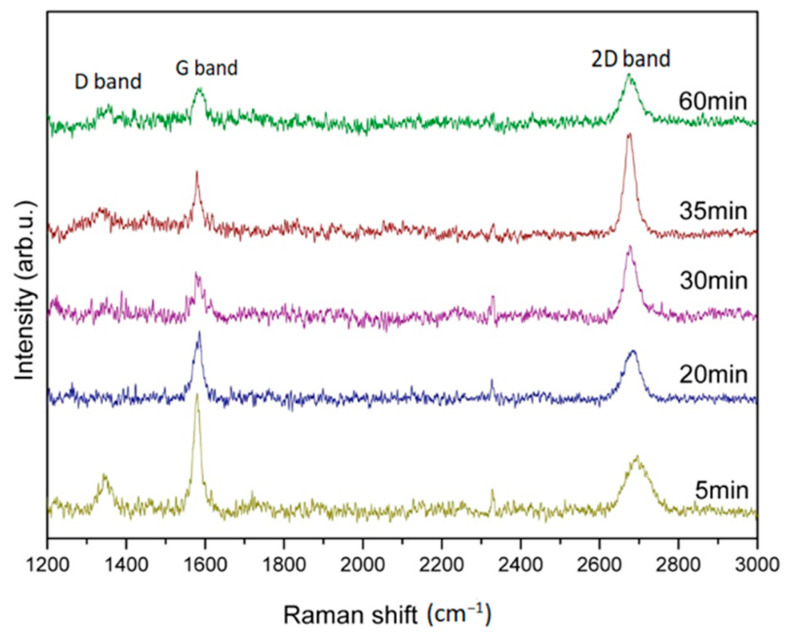
Raman spectra of graphene grown with optimal H_2_/Ar gas flow compositions of 15/20 sccm and temperature of 900 °C in different time duration of 5, 20, 30, 35, and 60 min., respectively.

Figure 6 shows Raman spectra for graphene growth with optimal H_2_/Ar gas flow compositions of 15/20 sccm, and suitable temperatures of 900 °C in different time durations of 5, 20, 30, 35, and 60 min., respectively. It can be clearly observed that all specimens with different growth times duration show the graphene characteristic peaks. The D band has also been found at ~1350 cm^−1^, whereas the synthesis period is in 5, 35, and 60 min. in which the I_D_/I_G_ is calculated as 0.33, 0.42, and 0.54, respectively. The D band indicates the disordered arrangement of graphene structures inside. No D band was observed at the synthesis time duration of around 20~30 min. indicating an optimal growth period. Moreover, either shortening or extending the process period will result in the degradation of graphene quality. The I_2D_/I_G_ ratio and 2D-FWHM value at different growth periods of 5, 20, 30, 35, and 60 min. were, respectively, calculated as 0.47, 0.62, 1.38, 1.60, and 1.43, and 66, 54, 36, 30, and 60 cm^−1^.

**Figure 7:**
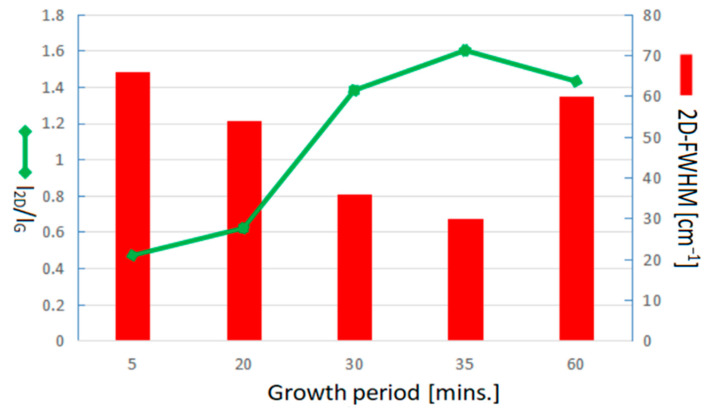
Comparison of I_2D_/I_G_ ratio and 2D-FWHM value of Raman pattern corresponding to the graphene growth period of 5, 20, 30, 35, and 60 min in accordance with Raman spectra of Figure 6.

Figure 7 plots a comparison of I_2D_/I_G_ ratio and 2D-FWHM value of Raman spectra corresponding to the graphene growth period of 5, 20, 30, 35, and 60 min. in accordance with Raman spectra of Figure 6 for more detailed discussions. Shorten growth period will produce few-layer graphene and beget more defects. Good quality graphene also can be found as the growth period extends to 35 min. The I_2D_/I_G_ ratio decreases slightly while the growth time further extends to 60 min. indicating a degradation of graphene quality because of the prolonged growth duration. Therefore, the growth period of around 30–35 min. completed the good quality of the few-layer graphene synthesized in this study. The growth period of around 30 min. for industrial application is our priority suggestion because of the resources, cost saving, and time efficiency.

**Figure 8:**
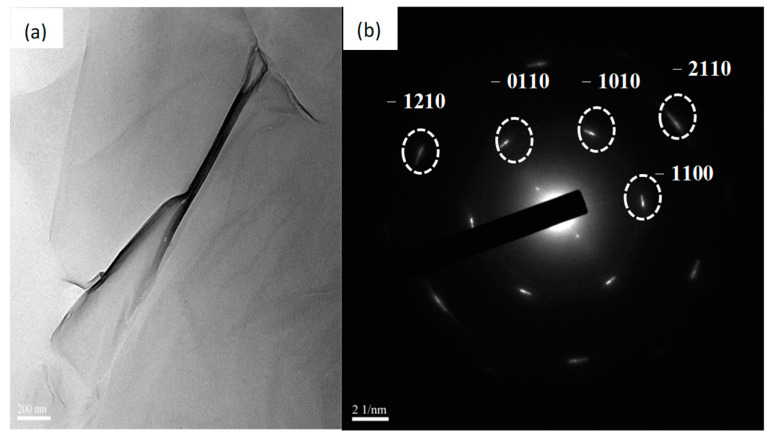
(**a**) TEM images and (**b**) corresponding electron diffraction pattern of graphene synthesized at optimal H_2_/Ar gas flow compositions of 15/20 sccm, temperature of 900 °C, and time duration of 35 min.

Further characterizing the graphene, TEM observation was involved. Figure 8 shows the structural features taken from the methanol precursor-based graphene synthesized at H_2_/Ar gas flow compositions of 15/20 sccm, temperature of 900 °C, and time duration of 30 min. Figure 8a depicts a TEM image of graphene. It can be seen that the as-grown graphene is with scrolling and corrugation. Figure 8b illustrates the electron diffraction pattern of graphene with a regular hexagonal diffraction pattern indicating the long-range crystalline ordering of more than one single-layer graphene film.

**Figure 9:**
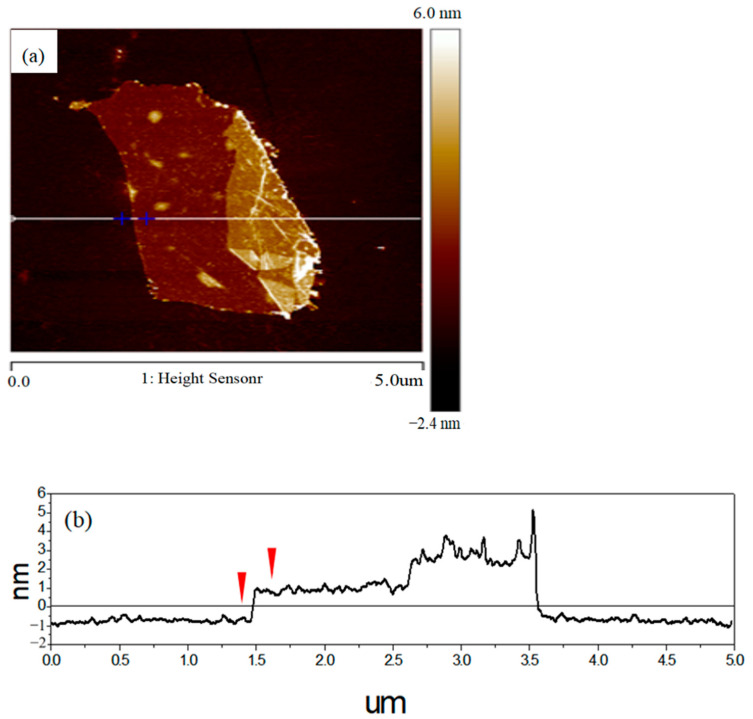
(**a**) AFM image of graphene on Si substrates and (**b**) corresponding height profile from the indicated white line and location. The location of anti-trigonometric indicator in Figure 9 (**b**) corresponds to the plus sign position of Figure 9 (**a**).

AFM is an alternative tool for measuring the thickness of graphene due to the 0.1 nm precision in *Z*-axis. Figure 9a shows the AFM image of graphene on Si substrates. Figure 9b depicts the height profile from the indicated white line and the location of Figure 9a. The AFM morphology observation shows that the graphene surface is layered stacking with a thickness of around 1.3–2.0 nm. it is well-known that the theoretical thickness of monolayer graphene is around 0.335 nm [58,59,60]. However, the practical measuring result could be thicker than the theoretical value due to the existence of adsorbates on the surface of the graphene. Therefore, the number of graphene layers prepared in this study can be speculated at about 2–4 layers.

In summary of the Raman spectra studies, few-layer graphene can be synthesized with the H_2_ gas flow rate fixing at 15 sccm in a lower growth temperature of 700 °C within 30 min or in a growth temperature of 900 °C within only 5 min. Graphene can’t be synthesized with both the lower temperature of 700 °C and a shorter time duration of 5 min indicating that few-layer graphene is hardly synthesized, saving both the thermal energy and time period. Moreover, few-layer graphene growth at a lower temperature of 700 °C within 30 min shows more defects than that at a temperature of 900 °C within only 5 min, indicating the choice of improvement. This defects observation also proved that the growth period can be saved by increasing the growth temperature.

It should be noted that at the beginning of the graphene growth process via the chemical vapor deposition method, blunt gas and reducing gas such as H_2_ and Ar are generally used to drive away the oxygen and impurities in the quartz tube and related pipelines of the system and further reduce the copper oxide on the surface of the copper foil substrate while the temperature is increasing to the reaction condition. In order to further discuss the function and role of pretreatment gas, the relationship between different pretreatment gas compositions and graphene formation has been investigated.

**Figure 10:**
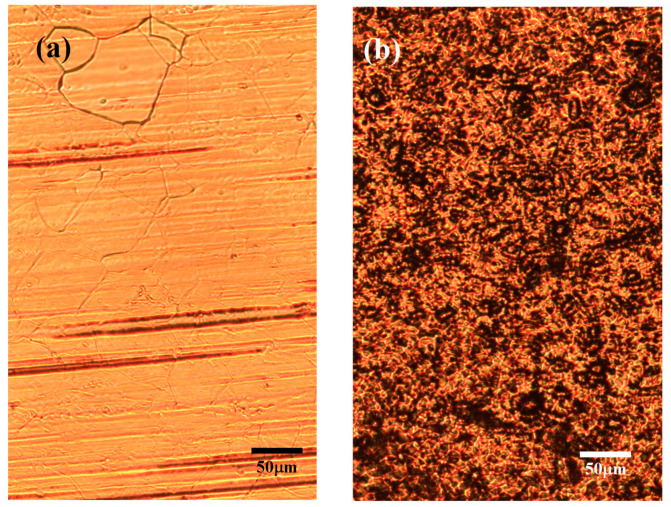
Optical photomicrographs of the copper foil substrate after optimal graphene growth with pretreatment atmosphere of (**a**) H_2_/Ar and (**b**) Ar compositions, respectively.

Figure 10 shows optical photomicrographs of the copper foil substrate treated with H_2_/Ar of 15/20 sccm carrying methanol at a growth temperature of 900 °C and a growth period of 30 min, with a pretreatment atmosphere of (a) H_2_/Ar and (b) Ar gas compositions. It can be seen in the optical microscopy photos that the copper foil surface is clear and shining with the H_2_/Ar atmosphere pretreatment, but many black dots are distributed on the copper foil surface with the pure Ar atmosphere pretreatment. These black dots are probably not the graphene formation but the deposition of contaminants on the copper foil surface because the shape of these black spots does not resemble the shape of a graphene hexagonal structure. For the confirmation of this feature, the copper foil substrates with stander graphene growth processes using different pretreatment gas of H_2_/Ar or Ar atmospheres have also been inspected via Raman spectroscopy measurement.

**Figure 11:**
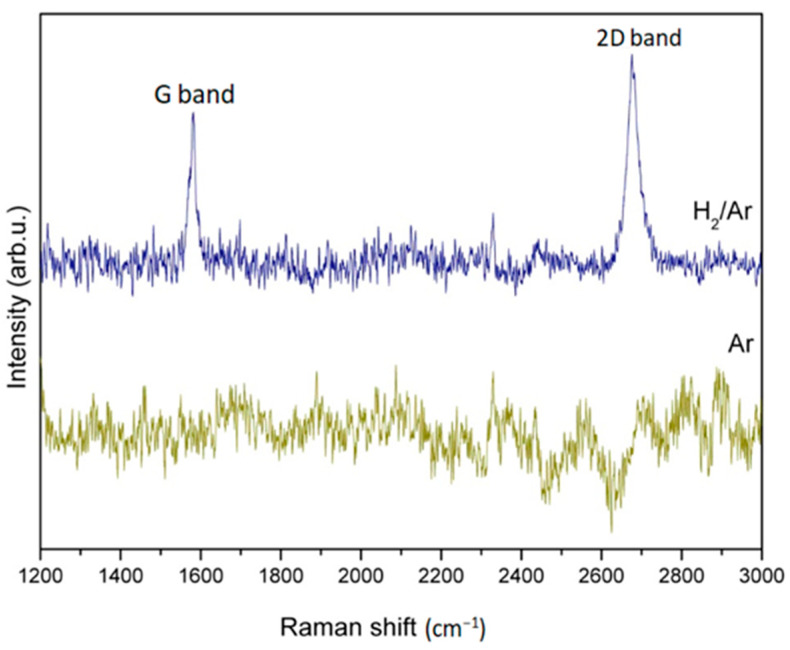
Raman spectroscopy pattern of the graphene grown on copper foil substrates after optimal graphene growth with pretreatment atmosphere of H_2_/Ar or Ar compositions, respectively.

Figure 11 shows the Raman pattern of the graphene corresponding to Figure 8 in different pretreatment atmospheres of H_2_/Ar or Ar. It is found that the G band and 2D band graphene characteristic peaks, respectively, appear at ~1600 cm^−1^ and ~2700 cm^−1^, when H_2_/Ar is used as the pretreatment gas.

However, no graphene characteristic peak is shown on copper foil substrates pretreated with pure Ar gas pretreatment. It can be deduced that possible contaminants on the surface of the copper foil can be removed by using H_2_/Ar as the pretreatment gas at high temperatures, which is conducive to graphene generation.

## 4. Conclusions

Optical microscopy observation, Raman spectra measurement, I_2D_/I_G_ ratio calculation, and 2D-FWHM value comparisons proved that few-layer of graphene had been successfully synthesized on copper foil with methanol as a carbon source by the chemical vapor deposition method. It is also found that the cost-efficient growth condition in H_2_/Ar atmosphere flow ratio, temperature, and time period are 15/20 sccm, 900 °C, and 30 min, respectively. A number of monolayer graphenes was also found in similar standard procedures, but they required higher growth temperatures and longer time periods. In addition, graphene can be synthesized without adding hydrogen gas flow because the H_2_ can be induced from the decomposition of methanol. With the H_2_ gas flow rate fixed at 15 sccm, few-layer graphene was synthesized at the lower growth temperature of 700 °C in 30 min, and at 900 °C growth temperature in only 5 min. Few-layer graphene growth in a lower temperature of 700 °C within 30 min. shows more defects than that in a temperature of 900 °C within only 5 min indicating that the quality of few-layer graphene can be improved by increasing the growth temperature without extending the growth periods. Moreover, pretreatment atmosphere composition choosing before graphene growth is a significant procedure because it may deduce a successful synthesis or not.

## Data Availability

Not applied.
